# Assessment of Knowledge, Attitude, and Practice on Safe Working in Confined Space among Male Water Services Workers in the Central Region of Malaysia

**DOI:** 10.3390/ijerph19127416

**Published:** 2022-06-16

**Authors:** Hamiza Ngah, Suhaily Mohd Hairon, Nurul Ainun Hamzah, Shahronizam Noordin, Mohd Nazri Shafei

**Affiliations:** 1 Department of Community Medicine, School of Medical Sciences, Universiti Sains Malaysia, Kubang Kerian 16150, Kelantan, Malaysia; drhamizangah@moh.gov.my (H.N.); suhailymh@usm.my (S.M.H.); 2 Environmental and Occupational Health Programme, School of Health Sciences, Universiti Sains Malaysia, Kubang Kerian 16150, Kelantan, Malaysia; nurulainun@usm.my; 3 National Institute of Occupational Safety and Health, Bangi 43650, Selangor, Malaysia; shahronizam@niosh.com.my

**Keywords:** confined space, workplace safety, knowledge, attitude, practice

## Abstract

Employee performance in terms of knowledge of job scope, safe working practices, and safety-related attitude at work are used to measure an organization’s success in managing employee welfare and safety to prevent workplace injury and death. This study aims to determine the level of knowledge, attitude, and practice on safe working in confined space among water services workers. A cross-sectional study involving a randomly selected 207 water services workers working in a confined space was performed in the central region of Malaysia. The assessment was performed using a validated Malay self-administered questionnaire. Descriptive statistics, Chi-square test and Logistic regression were used for data analyses. The study’s participants were all men, with a mean (SD) age of 35.2 (8.83) years. The average working duration was 10.8 years. Overall, 67.1% of participants had good knowledge, while 65.7% had a positive attitude. The majority of the workers (60.4%) were found to follow safe working practices. Regression analysis revealed that significant predictors for knowledge were age [Adjusted odds ratio (Adj. OR) 2.793; 95% CI: 1.310, 5.955; *p* = 0.008] and attitude (Adj. OR 2.127; 95% CI: 1.011, 4.526; *p* = 0.048). Attitude was influenced by marital status (Adj. OR 4.126; 95% CI: 2.079, 8.186; *p* < 0.001) and knowledge level (Adj. OR 2.224; 95% CI: 1.025, 4.824; *p* = 0.043). A positive attitude was the sole predictor influencing the safe practice (Adj. OR; 1.878; 95% CI: 1.041, 3.388; *p* = 0.036). In conclusion, the workers’ levels of knowledge, attitudes, and practices were relatively satisfactory. Extensive investment in workplace safety and health programs, appropriate training, growth opportunities, and effective employee performance evaluation methodologies may assist workers in performing at their best.

## 1. Introduction

Working in confined spaces (CS) is very risky and carries potential hazards because such spaces are not primarily designed or intended for continuous human occupancy [1]. A confined space is a workplace that poses a risk of injury to anybody who works there; as a result, there are rigorous rules in place such as the Industry Code of Practice for Safe Working in Confined Space (ICOP) 2010, Occupational Safety and Health Act (OSHA) 1994, and Factories and Machinery (Safe, Health and Welfare) Regulations, 1970. These are to ensure the safety of employees and other assets. While working in CS, failing to follow standard operating procedures may result in property damage as well as potentially fatal injury or disease [2]. Some CS like storage tanks, silos, sewers, and wells are easy to identify; however, other spaces like chambers or pipes might be less obvious but equally dangerous. Explosion or fire threats, high dust concentrations, a lack of oxygen, and high temperatures leading to a severe increase in body temperature can all make confined space unsafe [3].

The Census of Fatal Occupational Injuries (CFOI) data produced by the US Bureau of Labour Statistics reported that 1030 people were killed or injured due to CS from 2011 to 2018 [4]. Singapore witnessed 18 CS-related deaths between 2004 and 2014, representing a mortality rate of 0.08 per 100,000 workers [3]. There were 45 fatal CS accidents in Malaysia between 2009 and 2019, primarily due to a lack of awareness or expertise, risk assessment documents, and safe working procedures [5]. In 2020, the Department of Occupational Safety and Health, Malaysia reported CS fatalities increased to 13 compared to 6 fatalities in the previous year. According to the Fatal Accident Circumstances and Epidemiology (FACE) report, municipal water, and sanitary services, which include cleaning and repairing tanks, septic tanks, and sewers, account for the majority of occupation-specific deaths. Other industries linked to CS mortality include construction (particularly water system and sewerage construction, which is done by individual trade contractors), manufacturing, agriculture, electric utilities, and transportation [6].

Working in confined spaces is risky, yet it is unavoidable in the water services business. As a result, it is vital to devise and practice a safe working technique for such areas. The water services industry is an important infrastructure in ensuring human health and the environmental protection. They play a critical role in providing healthy clean water access to the society. As time goes on, environmental and health safety issues become more of a worry [7]. The nature of the tasks that confined space workers in the water industry must perform, such as cleaning of sludge and other waste materials, an inspection of the physical integrity of process equipment, maintenance, repair, including welding, modification, and adjustments to mechanical equipment, and construction purposes, necessitates complete attention [1].

As the sole water services provider in the central region of Malaysia, they are responsible to abstract, treat and distribute clean and safe water supply to 8.4 million consumers. All of their 34 water treatment plants operate 24 h every day to produce an average of 5000 million litres per day of treated water that is then distributed through over 29,000 km pipe length. It is their responsibility to perform frequent scheduled maintenance and cleaning work [7]. Workers inside the chamber must also conduct regular inspections to ensure the presence of chlorine can be safely maintained. Once a month, the sludge extraction system was cleaned. The accelerator’s raw water inlet was cleaned once or twice a month, potentially exposing workers to hazardous atmospheres, and making entry and exit difficult [8]. The total workforce required during the cleaning process is determined by the work conditions and duration. When cleaning and maintenance work must be done from day to night, two shift workers are involved.

Individuals must be appropriately trained and instructed on safety issues and how to apply them. A competent individual should perform a risk assessment of the work and working conditions inside CS, and then use the findings to identify the necessary precautions to reduce the hazardous conditions. Compulsory occupational safety and health training help workers improve their knowledge, skills, and attitudes [9,10]. A study conducted by Grau et al. (2002) on 140 tile Spanish workers demonstrates that safety training is linked to safety attitudes. Both safety standards and workers’ individual responsibility for safety were potential targets of safety attitudes [10]. However, for the training to be effective and capable of improving worker attitudes, certain quality conditions must be met [11]. This coincides with results from Aziz and Osman (2019) study, who found that effective training, as measured by learning performance and training transfer, does help to increase the participants’ knowledge, skills, and attitude. This is also in line with the occupational safety and health (OSH) model proposed by Christian et al. (2009), which states that occupational accidents and injuries can be reduced by creating a suitable OSH environment and providing employees with the necessary knowledge and motivation to improve safety compliance and participation [12].

Besides training, work experience as part of human capital also play a role in developing a good work safety culture that may impact on the quality of task completion, interactions and deliverables. Research on the relationship between work experience and KAP has been consistently conducted. The result of a study on among meat-handler workers found that workers with higher educational levels and longer work experience were more likely to obtain good knowledge, attitude and practice [13]. According to a study conducted by Salthouse (1997), the positive effects of job experience can be directed towards job performance.

The trinity of knowledge, attitude, and practice (KAP) is inextricably linked. They are directly proportional to each other [14]. The KAP survey is conducted for planning, implementing, and evaluating interventional measures. It is a method to collect information concerning people’s knowledge, understanding, and preconceived thoughts and ideas. In addition, the survey records how people demonstrate their knowledge and attitude through behaviour [15]. Workers’ knowledge, attitude, and practice concerning safe working in confined spaces can be used to develop safety prevention programs and training materials. It is impossible to have complete control over the working environment.

Hazards can be prevented by providing a safe working environment by creating and managing employee safety awareness, workplace safety climate, and organizational safety culture [16]. As Westaby and Lee (2003) pointed out, safety consciousness entails awareness and a positive attitude toward safety. Safety consciousness affects safety-related activity, self-esteem, and organizational support concerning hazard occurrence [17]. Worker safety consciousness is directly influenced by risk perception, job satisfaction, and organizational support [18].

The main research aim for this study was to determine the level of knowledge, attitude, and practice among male water service workers in the Central Region of Malaysia regarding safe working in confined spaces, as well as the associated factors. Finally, a statistical model was created to assess the acceptable degree of workers’ knowledge, attitude, and practise when working in a restricted area, which would subsequently be used to support the database on confined space from a local perspective.

## 2. Materials and Methods

### 2.1. Study Design and Subjects

A cross-sectional study was conducted between October to December 2020 in the central region of Malaysia which involved Kuala Lumpur, Selangor, and Putrajaya. Water service workers who work in confined spaces participated in this study. The administrative clearance was secured from the Head of Department, meanwhile, the employee roster was received from the Water Quality Services Department with the help of the Health, Safety, and Environmental officer.

The sample size was calculated using a single mean formula. The estimate was based on a 95% confidence interval of 57% positive attitude towards workplace health and safety [15]. The study required 130 subjects determined using a type 1 error (alpha) of 0.05 (two-tailed), 80% power of the study, and an expected 20% non-response rate. A simple random sampling method using a random number generator function was applied to obtain the final list of participants.

Based on the study’s eligibility criteria which were: (1) permanent or contract-based confined space workers having 8.00 a.m. to 5.00 p.m. office hours; (2) 18 years or older, holding more than six months experience to ensure adequate engagement at work; (3) able to read and understand Bahasa Malaysia, 264 individuals agreed to participate in the survey. Participants were briefed about the study objective and given written consent before data collection.

### 2.2. Data Collection

The data was collected using a validated Malay version of the Knowledge, Attitude, and Practice on Safe Working in Confined Space Questionnaire (CS-KAP) [19]. It consists of 49 questions that cover demographic information, health state, occupational information, knowledge, attitude, and workplace practice. There are seven, five, and 10 items in the knowledge, attitude, and practice sections, respectively. Cronbach’s alpha values for knowledge, attitude, and practice-based questions were 0.631, 0.804, and 0.917, respectively. Raykov’s rho internal consistency reliability values were 0.814 and 0.912 for attitude and practice questionnaires, respectively. The average time it took to complete a questionnaire was 15 min.

The knowledge questionnaire covers the basics of safe CS work, including equipment and hazard-specific aspects. The number of correct responses per item was used to score respondents. Each correct response received a score of one, while a wrong or unsure response received a score of zero. Questions on safe working procedures and health surveillance were included in the attitude section. The section was evaluated using a five-point Likert scale ranging from 5 (strongly agree) to 1 (strongly disagree). A five-point Likert scale ranging from ‘strongly agree’ to ‘strongly disagree’ was used because it was most recommended by the researchers to reduce patient respondents’ frustration and increase response rate and response quality [20,21,22] Items that were negatively stated were recorded, with a higher score indicating a good attitude and a lower score indicating a negative attitude. Questions about CS risk prevention and personal protective equipment (PPE) were addressed in the practices section. A four-point Likert scale ranging from 1 (never) to 4 (always) was used in the section. For safe behaviours, scores of “4”, “3”, “2”, and “1” were assigned for replies of “always”, “often”, “seldom”, and “never”, respectively. On the other hand, the scoring system was reversed for unsafe practice.

During data collection, we were assisted by the health, safety, and environmental officer. CS workers were identified and gathered at three separate water treatment plants based on implementation zone divisions. During each data collection session, participants were briefed on the purpose of the study, questionnaire structure, and ethical concerns. Each participant signed a written consent form. The participants were provided with a self-administered questionnaire and were given adequate time to complete it. They were also given the option of submitting the questionnaire once it was done. At the end of the data collection session, all participants were given a token of appreciation for their participation.

### 2.3. Statistical Analysis

The data were extracted, converted to Microsoft Excel format, and then exported to SPSS version 26. Exploratory data analysis was performed to check for missing data and assess the distribution of numerical variables. Numerical data were presented as mean (SD) or median (IQR) based on normality distribution. Categorical data were presented as number (*n*) and percentage (%). The mean (SD) for each KAP item was also analysed. The scores for knowledge, attitude, and practice were transformed into percentage scores by dividing the participant scores by the maximum scores and multiplying by 100. Two categories were used to record knowledge, attitude, and practice: (1) Knowledge (good or bad), (2) Attitude (positive or negative), and (3) Practice (safe or unsafe).

The z-scores below the mean have a poor negative value, and those above the mean have a positive value [23]. The cut-off point for knowledge, attitude and practice were 78, 90 and 80 respectively. It was used to categorise the good and poor knowledge, positive and negative attitudes, as well as safe and unsafe practices. Chi-square (χ^2^) test was used to analyse the association between two categorical variables (knowledge vs. attitude, knowledge vs. practice, attitude vs. practice). Logistic regression analysis was used to determine the associated factors influencing the outcome variables (good knowledge, positive attitude, and safe practice) among participants. Variables with *p* < 0.25 were selected to be included in the multivariable analysis. The final model was presented as a crude and adjusted odds ratio (Adj. OR), regression coefficient (B), and *p*-value. The significant level was set at *p* < 0.05.

## 3. Results

Out of the total 264 workers recruited in the study, 207 workers were able to take part in the survey, giving a response rate of 79.6%. All of the participants were male, with a mean age (Standard Deviation (SD)) of 35.2 (8.83) years old. The majority of the participants were Malay (94.7%), married (77.3%), and had completed tertiary education (52.7%). Concerning employment status, the mean (SD) working duration was 10.8 (7.77), while the median (IQR) CS experience duration was 9.0 (9.17) years. Multitasking was essential for most workers’ jobs, such as cleaning and maintenance. More than half had received CS training (58.5%) and attended toolbox meetings before working (87.0%). The majority of them always use a safety shoes/boot (89.4, *n* = 185), safety helmet (85.0%, *n* = 176), safety gloves (77.8%, *n* = 161), and eye protection (61.4%, *n* = 127) while working.

The majority of participants correctly answered almost every question on the knowledge questionnaire except questions concerning confined space ventilation that should be placed at the beginning of the confined space only when work is carried out (K5). Question K1, which dealt with occupational risk assessment, had the highest percentage of accurate answers (97.1%), with participants correctly stating that an assessment must be completed before workers enter a confined space. Participants’ symmetrical option responses are shown in Table 1.

More than half of the CS workers strongly agree on A4: campaigns for occupational health and safety are an effective way to promote and educate workers and A5: the priority of occupational health and safety at the workplace. Table 2 shows the findings on attitudes toward working in a confined space.

Table 3 shows the descriptive statistics of the practice section concerning safe working in a CS. Less than half of the participants answered “always” on wearing the body harness (36.2%), ear protection (37.7%), reflective safety jacket (44.4%), and respiratory protection (46.4%) when working in confined spaces. Most workers, 162 (78.3%), use a safety helmet when working in CS.

The total mean (SD) percentage scores for knowledge, attitude, and practice were 78.1 (15.91), 92.7 (8.53), and 80.2 (19.09), respectively. The mean score for each KAP was used as a benchmark for satisfactory and unsatisfactory levels, respectively. Those who scored 78% or more were considered to have good knowledge. Table 4 illustrates that 139 (67.1%) of the workers had good knowledge on safe working in CS. In the attitude and practice sections, those who scored 92% and 80% or more were considered to have a positive attitude and follow safe practices while working in CS. There were 136 (65.7%) workers who had a positive attitude toward safe working in CS, whereas 125 (60.4%) workers practised safe working in CS.

Chi-square analysis showed that there were statistically significant associations between knowledge with attitude level (χ^2^(1) = 5.484, *p* = 0.019) and attitude with practice level (χ^2^(1) = 4.235, *p* = 0.040). However, there were no significant associations between knowledge and practice level (χ^2^(1) = 0.655, *p* = 0.418). This study found that 119 confined space workers (87.5%) had a positive attitude when they were knowledgeable concerning safe working in CS. The majority of the workers (71.2%) had a positive attitude toward safe practice (*n* = 89) while 35 (42.7%) of them had a negative attitude toward unsafe practice. There was 80.5% (*n* = 66) of workers who were knowledgeable about safe working in CS had unsafe practices. A total of 106 (84.8%) workers had good knowledge and safe practice.

Univariable logistic regression analysis was performed for an outcome of good knowledge and found that age ≥ 30 years old, higher education level, married, working duration ≥ 5 years, CS experience duration ≥ 2 years, and positive attitude had a *p*-value of <0.25. As such, these variables were included in the multivariate model as displayed in Table 4. Age ≥ 30 years old (Adj. OR 2.793; 95% CI: 1.310, 5.955; *p* = 0.008) and positive attitude workers (Adj. OR 2.127; 95% CI: 1.011, 4.526; *p* = 0.048) were identified significantly associated with good knowledge when adjusted for all these variables. In terms of association with attitude level, the statistically significant result was good knowledge and married workers. The workers with good knowledge had 2.224 times the odds compared to workers with poor knowledge to have a positive attitude (95% CI: 1.025, 4.824, *p* = 0.043) when adjusted for age, marital status, education status, working duration, and practice level. Married were more likely to have a positive attitude than those who were unmarried (Adj OR 4.126; 95% CI: 2.079, 8.186; *p* < 0.001). As for practice level, two variables (positive attitude and ICOP briefing) were included in the final model testing of logistic regression which showed that only those who had a positive attitude had a significant association with the safe working practice (Adj. OR 1.878; 95% CI: 1.041, 3.388; *p* = 0.036) when adjusted for education status, marital status, and ICOP briefing (Table 4).

## 4. Discussion

There is a lack of scientific information on safe working knowledge, attitude, and practice (KAP) among confined space workers in Malaysia. Therefore, KAP questionnaire on safe working in confined spaces was developed according to the Industrial Code of Practice Confined fine Space in Malaysia. KAP assessments of the workers should focus on following safety recommendations to reduce workplace accidents and injuries. It also assists in the collection of data to assist management in identifying impediments to worker safety and assisting the target population in taking preventive steps [24,25].

The participants in this study are all men. As working in a confined space reinforces masculine norms, making it difficult for women to succeed, men are dominating [26]. Furthermore, because CS is a male-dominated field, it limits women’s willingness to join, placing them at a disadvantage [27]. The mean (SD) working duration was 10.8 (7.77) years, while the median (IQR) duration of working in CS was about 9.0 (9.17) years. Because the questions are related to how they work daily, respondents with this experience are more likely to give better answers.

In the present study, most workers were aware of the need for occupational risk assessment before entering the CS. Occupational risk assessment involves hazard identification, risk assessment and risk control (HIRARC), which is an initial activity comprising the occupational health and safety management system [27]. It should be conducted primarily to support the decision-making process regarding workplace safety and health [28].

The reversed statement on the knowledge question (item K5) revealed that about 83% of the participants did not know that ventilation in the confined space should be used throughout CS work rather than simply at the start. Some workers might use ventilation to clear the contaminated air inside the CS and then turn it off once the air is cleaner [29]. Mechanical ventilation is an effective tool for lowering exposure levels. Throughout the working process, however, the supply and exhaust hoods must be properly placed and positioned at the fume generation point [30]. However, if there is no information available on the required configuration, the required ventilation time before entry, and the continuous ventilation requirement for air quality control is given, some workers may have difficulties with the ventilation that is supposed to exist in a confined space area [31]. In the present study, observations of the study location revealed that technical ventilation calculation documents for the water services industry are accessible. Another explanation for the high percentage (83.6%) for the wrong answer on item K5 could be participants’ confusion and lack of attention despite indicating that a reverse statement was introduced to prevent response bias [32].

The majority of the respondents strongly agree that workplace safety is everyone’s responsibility. Workplace safety includes things like ensuring occupational health and safety remains the top priority when doing CS work, transparency regarding entry permits, periodic health surveillance for every CS worker, and occupational health and safety campaigns at the workplace. The permit issuer must have information about the CS, the work to be performed, and the work environment to complete the risk assessment correctly. This ensures that risks are neither overlooked nor underestimated [31].

In this study, 200 (96.6%) workers used PPE during work. In comparison through studies conducted in Western countries, the utilization of PPE in our population was observed to be higher [33,34,35]. It has been demonstrated that awareness of the use of PPE increases when there is a sense of responsibility for their employees’ wellbeing and safety in the workplace. The willingness and obligation of the employer to give PPE training, safety training, and safety orientation before starting work, as well as the presence of supervision, may all contribute to the effective use of PPE [36]. A study concerning small industry workers in Jeddah indicated that glove and ear protection use was even lower than in the present study [37]. However, there is diversity in the types of PPE used by the workers. Differences in PPE usage can partly be attributed to a lack of awareness and provision of when PPE is required, what equipment is required, how to use or wear it, and which PPE they should most importantly utilize. Furthermore, boots, masks, gloves, and goggles are examples of PPE that might pose issues in terms of comfort and durability, especially when compounded by working in ill-fitting environments like confined spaces [38].

The present study shows that water services workers had a good understanding on safe working in CS, where more than half of workers managed to achieve a satisfactory score on the KAP questionnaire. A study by Mukhtar et al. [39] concerning workers in petrochemical companies indicated almost similar results, where most of the workers had a high degree of knowledge (95.7%), a positive attitude (70.0%), and fair practices (50.0%) on occupational safety and health. The highest percentage of workers who completed CS training and attended toolbox meetings concerning specific tasks might be responsible for this observation. A training program can strengthen employee skills and enhance knowledge and attitude [40]. The mandatory general training scheme for all workers with job-specific information assists organizations to build safer human capital at scale [19]; this is why most countries, including Malaysia, require CS workers to attend safety training before starting CS work [41,42].

The Industry Code of Practice for Safe Working in a Confined Space 2010 (ICOP) provides detailed information concerning safety and health for people who need to enter or work in CS [42]. Proper information delivery and a productive management team benefit an organization by fostering a positive attitude [43]. In the present study, workers were 30 years old and older, and having a positive attitude was found to be significantly associated with having good knowledge. As suggested by Gyekye et al. [44], older workers had a higher level of job satisfaction and a better view of safety. They discovered that older workers were the most compliant with safety protocols and had the lowest risk of being involved in an accident. In addition, as you get older, the more work experience you gained [45]. Furthermore, good relationships between the employer and employees foster a positive and correct attitude [43].

This study discovered those who had good knowledge and were married were noted to have an impact on a positive attitude toward safe working in CS. Those who have a positive attitude display safe practice. The most likely explanation is that marriage entails greater obligations, which may make stable employment more valuable and important. At work, job satisfaction and organizational commitment have the greatest potential to influence how we behave [46]. Giving workers a sense of belonging to the organization would likely encourage them to be more engaged, more motivated, and more likely to perform at a high level with safety as their priority [47].

However, the present study found that knowledgeable workers showed no association with their practice. The percentage of those who were knowledgeable about safe practices and those who were knowledgeable but had unsafe practices were approximately the same. This state indicates an urgent need for continuous monitoring and supervision from the organization to ensure workers comply with the guidelines to avoid or minimize occupational accidents. According to Zahiri Harsini and colleagues [48], poor direct safety management and monitoring, managers’ lack of authority and power, and a lack of specific funding for workplace safety all contributed to unsafe behaviour and mistakes occurrences. Similarly, a study was done by Naghavi et al. [49] also found that unsafe acts might be provoked by ineffective team and resource management efforts as well as a lack of supervision concerning health, safety, and the environment. Furthermore, poor working environments were influencing workers’ ability to safely practice occupational safety at the workplace [50]. Inadequate staffing may also put workers at risk of occupational accidents due to job overload and exhaustion, which may drive them to make mistakes that are dangerous to themselves and their coworkers [50,51].

The need for adequate and up-to-date safety procedures as well as effective safety intervention should be highlighted to empower the workers’ awareness of safe practices in the workplace. Safety responsibility and awareness should come from an individual to reduce and eliminate risk factors for unsafe practices. However, management must offer adequate resources for safety and be willing to invest more in safety to foster workers’ commitment to safety.

## 5. Conclusions

The knowledgeable workers were aware of safety while working in CS. However, despite having good knowledge of safe work in CS, they demonstrated unsafe practie. Nonetheless, the majority of workers possessed good knowledge, positive attitudes, and safe practises, which aided in reducing workplace hazards and assisting the organisation in meeting the challenges ahead. Confined space workers in the water service industry appear to adhere to safe working practices in CS as mandated by ICOP. The findings of this study provide evidence for decision-makers and policymakers in Malaysia to identify obstacles and advocate for the effective adoption of a safe working environment and practice. Furthermore, this study serves as a starting point for public health physicians or occupational health doctors to prioritize occupational health and safety in order to reduce or eliminate workplace hazards. Continuous training programs on safe work in CS as well as audits and supervision from top management, can help to ensure workers consistently achieve good performance and would translate what they have learned into practice. The good performance of workers could be assessed by decreased accident rate, decreased work process costs or decreased employee turnover.

## Figures and Tables

**Table 1:** Descriptive statistics of knowledge on safe working in a confined space among water services workers (*n* = 207).

Item	Result [*n* (%)]	
	Correct	Incorrect
K1 Occupational risk assessment (Hazard identification, risk assessment and risk control-HIRARC) must be done before the entry of workers in confined spaces)	201(97.1)	6 (2.9)
K2 Employers need to ensure that warning signs “DANGER-CONFINED SPACE. NO ENTRY” is placed near the entrance of the confined spaces	197 (95.2)	10 (4.8)
K3 Confined space workers are exposed to hazardous gases within the scope of the workplace	185 (89.4)	22 (10.4)
K4 Confined space workers must have confined space entry training recognized by the Department of Occupational Safety and Health	181 (87.4)	26 (12.6)
K5 Ventilation in the confined space should be placed at the beginning of the confined space work only when work is carried out	34 (16.4)	173 (83.6)
K6 Exhaust from any equipment placed near a confined space is the cause of the existence of a hazardous atmosphere in the confined space	157 (75.8)	50 (24.2)
K7 Difficulty breathing is a sign of exposure to hazardous atmosphere when working in a confined space	179 (86.5)	28 (13.5)

**Table 2:** Descriptive statistics of attitude on safe working in a confined space among water services workers (*n* = 207).

Item	Response [*n* (%)]					Min, Max	Mean (SD)
	Strongly Disagree	Disagree	Not Sure	Agree	Strongly Agree		
A1 I believe employees and employers are fully responsible for the safety of employees in the workplace	1 (0.5)	1 (0.5)	2 (1.0)	51 (24.6)	152 (73.4)	1, 5	4.70 (0.56)
A2 I believe the entry permit to the confined space needs to be informed and explained to the employees before the confined space work is carried out	1 (0.5)	0(0.0)	5 (2.4)	44 (21.3)	157 (75.8)	1, 5	4.72 (0.56)
A3 I think the health check-ups of confined space workers should be done periodically	0 (0.0)	1 (0.5)	8 (3.9)	84 (40.6)	114 (55.1)	2, 5	4.50 (0.60)
A4 I believe occupational health and safety campaigns are an effective way to promote and educate employees	0 (0.0)	0 (0.0)	6 (2.9)	79 (38.2)	122 (58.9)	3, 5	4.56 (0.55)
A5 Occupational health and safety are my top priority when I do the confined space work	0 (0.0)	0 (0.0)	4 (1.9)	57 (27.5)	146 (70.5)	3, 5	4.69 (0.51)

**Table 3:** Descriptive statistics of practice on safe working in a confined space among water services workers (*n* = 207).

Item	Response [*n* (%)]				Min, Max	Mean (SD)
	Never	Seldom	Often	Always		
P1 I make sure the situation in the confined space is safe before entering the confined space	15 (7.2)	11 (5.3)	25 (12.1)	156 (75.4)	1, 4	3.56 (0.89)
P2 I check all safety equipment and work tools are in a safe condition to use	14 (6.8)	13 (6.3)	27 (13.0)	153 (73.9)	1, 4	3.54 (0.89)
P3 I tell the employer if the safety equipment to do the work in the confined space is incomplete	12 (5.8)	15 (7.2)	24 (11.6)	156 (75.4)	1, 4	3.57 (0.86)
P4 I wear safety gloves while handling work in confined spaces	13 (6.3)	25 (12.1)	41 (19.8)	128 (61.8)	1, 4	3.37 (0.93)
P5 I wear a safety helmet when handling work in a confined space	12 (5.8)	13 (6.3)	20 (9.7)	162 (78.3)	1, 4	3.60 (0.85)
P6 I wear eye protection when handling work in a confined space	18 (8.7)	32 (15.5)	38 (18.4)	119 (57.5)	1, 4	3.25 (1.01)
P7 I wear ear protection when handling work in a confined space)	47 (22.7)	47 (22.7)	35 (16.9)	78 (37.7)	1, 4	2.70 (1.19)
P8 I wear respiratory protection while handling work in a confined space	41 (19.8)	32 (15.5)	38 (18.4)	96 (46.4)	1, 4	2.91 (1.19)
P9 I wear a body harness while handling work in a confined space	48 (23.2)	43 (20.8)	41 (19.8)	75 (36.2)	1, 4	2.69 (1.19)
P10 I wear a reflective safety jacket while handling work in a confined space	37 (17.9)	41 (19.8)	37 (17.9)	92 (44.4)	1, 4	2.89 (1.16)

**Table 4:** Factors associated with KAP towards safe working in a confined space by simple and multiple logistic regression analyses (*n* = 207).

Variables	KAP Level		Simple Logistic Regression		Multiple Logistic Regression		
			Crude OR (95% CI)	*p*-Value	B	Adj. OR (95%CI)	*p*-Value
	^a^ Knowledge Level						
	Good [*n* (%)]	Poor [*n* (%)]					
Age (year)							
<30	57 (73.1)	14 (10.9)	1			1	
≥30	115 (89.1)	21 (26.9)	3.026	0.004	1.107	2.793	0.008
			(1.434, 6.388)			(1.310, 5.955)	
Education status							
Up to secondary level	77 (78.6)	21 (21.4)	1				
Tertiary or higher level	95 (87.2)	14 (12.8)	1.851	0.103			
			(0.883, 3.879)				
Marital status							
Unmarried	37 (77.1)	11 (22.9)	1				
Married	135 (84.9)	24 (15.1)	1.672	0.208			
			(0.751, 3.725)				
Department							
Operation	53 (82.8)	11 (17.2)	1				
Production	119 (83.2)	24 (16.8)	1.029	0.943			
			(0.470, 2.253)				
Working duration (year)							
<5	42 (71.2)	17 (28.8)	1				
≥5	130 (87.8)	18 (12.2)	2.923	0.005			
			(1.383, 6.180)				
CS experience (year)							
<2	28 (71.8)	11 (28.2)	1				
≥2	144 (85.7)	24 (14.3)	2.357	0.041			
			(1.038, 5.354)				
CS training							
No	70 (81.4)	16 (18.6)	1				
Yes	102 (84.3)	19 (15.7)	1.227	0.583			
			(0.591, 2.550)				
Toolbox meeting							
No	21 (77.8)	6 (22.2)	1				
Yes	151 (83.9)	29 (16.1)	1.488	0.432			
			(0.553, 4.006)				
ICOP briefing							
No	34 (79.1)	9 (20.9)	1				
Yes	138 (84.1)	26 (15.9)	1.405	0.431			
			(0.603, 3.273)				
Attitude							
Negative	53 (74.6)	18 (25.4)	1			1	
Positive	119 (87.5)	17 (12.5)	2.377	0.021	0.755	2.127	0.048
			(1.137, 4.971)			(1.011, 4.526)	
Practice							
Unsafe	66 (80.5)	16 (19.5)	1				
Safe	106 (84.8)	19 (15.2)	1.352	0.419			
			(0.650, 2.814)				
	^b^ Attitude Level						
	Positive [*n* (%)]	Negative [*n* (%)]					
Age (year)							
<30	45 (57.7)	33 (42.3)	1				
≥30	91 (70.5)	38 (29.5)	1.756	0.060			
			(0.976, 3.160)				
Education status							
Up to secondary level	60 (61.2)	38 (38.8)	1				
Higher	76 (69.7)	33 (30.3)	1.459	0.199			
			(0.820, 2.595)				
Marital status							
Unmarried	19 (39.6)	29 (60.4)	1			1	
Married	117 (73.6)	42 (26.4)	4.252	<0.001	1.417	4.126	<0.001
			(2.160, 8.371)			(2.079, 8.186)	
Department							
Operation	40 (62.5)	24 (37.5)	1				
Production	96 (67.1)	47 (32.9)	1.226	0.517			
			(0.663, 2.266)				
Working duration (year)							
<5	34 (57.6)	25 (42.4)	1				
≥5	102 (68.9)	46 (31.1)	1.63	0.124			
			(0.875, 3.039)				
CS experience (year)							
<2	27 (69.2)	12 (30.8)	1				
≥2	109 (64.9)	59 (35.1)	0.821	0.607			
			(0.388, 1.739)				
CS training							
No	55 (64.0)	31 (29.5)	1				
Yes	81 (66.9)	40 (33.1)	1.141	0.655			
			(0.639, 2.040)				
Toolbox meeting							
No	19 (70.4)	8 (29.6)	1				
Yes	117 (65.0)	63 (35.0)	0.782	0.584			
			(0.324, 1.887)				
ICOP briefing							
No	29 (67.4)	14 (32.6)	1				
Yes	107 (65.2)	57 (34.8)	0.906	0.787			
			(0.444, 1.851)				
Knowledge							
Poor	17 (48.6)	18 (51.4)	1		1		
Good	119 (69.2)	53 (30.8)	2.377	0.021	0.799	2.224	0.043
			(1.137, 4.971)			(1.025, 4.824)	
Practice							
Unsafe	47 (57.3)	35 (42.7)	1				
Safe	89 (71.2)	36 (28.8)	1.841	0.041			
			(1.026, 3.302)				
	^c^ Practice Level						
	Safe [*n* (%)]	Unsafe [*n* (%)]					
Age (year)							
<30	46 (59.0)	32 (41.0)	1				
≥30	79 (61.2)	50 (38.8)	1.099	0.747			
			(0.619, 1.951)				
Education status							
Up to secondary level	53 (54.1)	45 (45.9)	1				
Higher	72 (66.1)	37 (33.9)	1.652	0.08			
			(0.943, 2.896)				
Marital status							
Unmarried	23 (47.9)	25 (52.1)	1				
Married	102 (64.2)	57 (35.8)	1.945	0.046			
			(1.013, 3.735)				
Department							
Operation	35 (54.7)	29 (45.3)	1				
Production	90 (62.9)	53 (37.1)	1.407	0.263			
			(0.774, 2.558)				
Working duration (year)							
<5	35 (59.3)	24 (40.7)	1				
≥5	90 (60.8)	58 (39.2)	1.064	0.843			
			(0.575, 1.969)				
CS experience (year)							
<2	23 (59.0)	16 (41.0)	1				
≥2	102 (60.7)	66 (39.3)	1.075	0.841			
			(0.529, 2.185)				
CS training							
No	53 (61.6)	33 (38.4)	1				
Yes	72 (59.5)	49 (40.5)	0.915	0.758			
			(0.519, 1.612)				
Toolbox meeting							
No	16 (59.3)	11 (40.7)	1				
Yes	109 (60.6)	71 (39.4)	1.055	0.898			
			(0.463, 2.406)				
ICOP briefing							
No	21 (48.8)	22 (51.2)	1		1		
Yes	104 (63.4)	60 (36.6)	1.816	0.084	0.624	1.866	0.074
			(0.923, 3.574)			(0.940, 3.704)	
Knowledge							
Poor	19 (54.3)	16 (45.7)	1				
Good	106 (61.6)	66 (38.4)	1.352	0.419			
			(0.650, 2.814)				
Attitude							
Negative	36 (50.7)	35 (49.3)	1		1		
Positive	89 (65.4)	47 (34.6)	1.841	0.041	0.630	1.878	0.036
			(1.026, 3.302)			(1.041, 3.388)	

^a^ Constant = 0.595; Forward LR method was applied; No multicollinearity and no interaction; Hosmer Lemeshow test, *p*-value = 0.851; Classification table 83.1% correctly classified; Area under ROC curve = 0.679 (95% CI: 0.583, 0.775); ^b^ Constant = −1.054; Backward LR method was applied; No multicollinearity and no interaction; Hosmer Lemeshow test, *p*-value = 0.157; Classification table 70.5% correctly classified; Area under ROC curve = 0.679 (95% CI: 0.600, 0.759); ^c^ Constant = −0.474; Backward LR method was applied; No multicollinearity and no interaction; Hosmer Lemeshow test, *p*-value = 0.963; Classification table 62.3% correctly classified; Area under ROC curve = 0.604 (0.569, 0.690).

## Data Availability

Not applicable.
